# Robotic vs. open partial cytoreductive nephrectomy in metastatic renal cell carcinoma: adverse in-hospital outcomes

**DOI:** 10.1007/s00345-025-06080-8

**Published:** 2025-11-11

**Authors:** Quynh Chi Le, Mattia Longoni, Andrea Marmiroli, Fabian Falkenbach, Calogero Catanzaro, Michele Nicolazzini, Federico Polverino, Jordan A. Goyal, Fred Saad, Riccardo Schiavina, Luca Fabio Carmignani, Alberto Briganti, Nicola Longo, Markus Graefen, Carlotta Palumbo, Miriam Traumann, Felix K.-H. Chun, Pierre I. Karakiewicz

**Affiliations:** 1 Cancer Prognostics and Health Outcomes Unit, Division of Urology, University of Montréal Health Center, Montréal, Québec Canada; 2 Department of Urology, University Hospital, Goethe University Frankfurt, Frankfurt am Main, Germany; 3 Division of Experimental Oncology/Unit of Urology, URI, Urological Research Institute, IRCCS San Raffaele Scientific Institute, Milan, Italy; 4 Vita-Salute San Raffaele University, Milan, Italy; 5 Department of Urology, IEO European Institute of Oncology, IRCCS, Via Ripamonti 435, Milan, Italy; 6 Università degli Studi di Milano, Milan, Italy; 7 Martini-Klinik Prostate Cancer Center, University Hospital Hamburg-Eppendorf, Hamburg, Germany; 8 Department of Urology, St. Orsola-Malpighi Hospital, University of Bologn, Bologna, Italy; 9 Division of Urology, Department of Translational Medicine, Maggiore della Carità Hospital, University of Eastern Piedmont, Novara, Italy; 10 Division of Urology, Department of Oncology, University of Turin, Orbassano, Italy; 11 Department of Neurosciences, Science of Reproduction and Odontostomatology, University of Naples Federico II, Naples, Italy

**Keywords:** Metastatic renal cell carcinoma, Partial cytoreductive nephrectomy, Robotic versus open surgical approach, Adverse in-hospital outcomes

## Abstract

**Objective:**

To test for adverse in-hospital outcomes after robotic (RPCN) vs. open partial cytoreductive nephrectomy (OPCN).

**Methods:**

RPCN and OPCN patients were retrospectively identified within the National Inpatient Sample database (2008–2019). Propensity score matching (PSM, ratio 1:2) and multivariable logistic regression models (LRM) were used.

**Results:**

Of 491 patients, 139 (28%) underwent RPCN vs. 352 (72%) OPCN. RPCN-rate increased from 4.2 to 42.5% over time (*p* < 0.001). RPCN patients exhibited similar age, comorbidity and race/ethnicity distribution relative to their OPCN counterparts. After 1:2 PSM, all 139 RPCN and 278 of 352 (79%) OPCN patients were included. Relative to OPCN, RPCN patients exhibited lower rates in four of 10 examined adverse in-hospital outcomes: intraoperative complications (< 3 vs. 9%, *p* = 0.02), pulmonary complications (6 vs. 14%, *p* = 0.02), blood transfusions (< 5 vs. 14%, *p* = 0.004) and exhibited shorter median length of stay (2 vs. 4 days, *p* < 0.001). In multivariable LRMs, RPCN independently predicted lower rates in the same four of 10 categories with odds ratio (OR) ranging from 0.17 to 0.34. Largest magnitude was recorded in shorter length of stay (OR 0.17, *p* < 0.001), followed by intraoperative complications (OR 0.24, *p* = 0.02), use of blood transfusions (OR 0.25, *p* = 0.003) and pulmonary complications (OR 0.34, *p* = 0.01). No differences in in-hospital mortality were recorded.

**Conclusion:**

Rates of RPCN has increased exponentially over time (4.2 to 42.5%). Relative to OPCN, RPCN is associated with fewer adverse in-hospital outcomes and shorter hospital stay. However, no differences regarding in-hospital mortality were recorded between RPCN and OPCN.

**Supplementary Information:**

The online version contains supplementary material available at 10.1007/s00345-025-06080-8.

## Introduction

Partial cytoreductive nephrectomy (PCN) represents a treatment option for patients with metastatic renal cell carcinoma (mRCC) [1–7]. It may either be performed using an open or robotic approach [3]. In general, robotic surgeries are associated with fewer adverse in-hospital outcomes compared to their open counterparts [8–10]. However, it is currently unknown whether robotic PCN (RPCN) offers an advantage over open PCN (OPCN) when adverse in-hospital outcomes represent the endpoint of interest. Moreover, the rates of RPCN are unknown.

To address this knowledge gap, we tested for differences in adverse in-hospital outcomes between RPCN and OPCN and assessed rates of RPCN. We hypothesized that rates of RPCN are increasing and important differences of adverse in-hospital outcomes may exist between RPCN vs. OPCN. To test these hypotheses, we relied on the National Inpatient Sample (NIS) database (2008–2019).

## Methods

### Data source and study population

The NIS is a set of longitudinal hospital inpatient databases included in the Healthcare Cost and Utilization Project (HCUP) and formed by the Agency for Healthcare Research and Quality (AHRQ) through a federal-state partnership [11]. All diagnoses and procedures were coded using the International Classification of Disease (ICD) 9th revision Clinical Modification (ICD-9-CM), ICD 10th revision Clinical Modification (ICD-10-CM), as well as ICD 10th revision Procedure Coding System (ICD-10-PCS). Due to the anonymous nature of the NIS, Human Ethics and Consent to Participate declarations was waivered.

### Study population

First, we identified patients aged ≥ 18 years with a primary diagnosis of RCC (ICD-9 code 189.0 and ICD-10 codes C64.1, C64.2, C64.9) with metastatic stage (Supplementary Table 1). Subsequently, patients who underwent PCN (ICD-9 codes 55.4 and ICD-10 codes 0TB00ZZ, 0TB03ZZ, 0TB04ZZ, 0TB07ZZ, 0TB08ZZ, 0TB10ZZ, 0TB13ZZ, 0TB14ZZ, 0TB17ZZ, 0TB18ZZ) were included and stratified according to RPCN vs. OPCN according to previously established methodology [12–15].

### Definition of variables for analyses

Study endpoints consisted of adverse in-hospital outcomes. These included overall complications, intraoperative complications, pulmonary, cardiac, vascular, gastrointestinal complications, as well as blood transfusions, length of stay (LOS), total hospital charges (THC) and in-hospital mortality. All characteristics were identified using ICD-9 and ICD-10 codes according to previously established methodology [12–15]. Comorbidities were defined according to Deyo modification of the Charlson Comorbidity Index (CCI) [16]. Covariates consisted of patient characteristics including age at admission (years, continuously coded), sex (male vs. female), race/ethnicity (Caucasian, African American, Others), CCI (0 vs.1–2 vs. ≥3) adjusted for metastatic disease, as well as hospital characteristics including region (Midwest, Northeast, South, West), teaching hospital status (teaching vs. non-teaching) and hospital bedsize (large [≥ 400 beds] vs. medium [200–399 beds] vs. small [< 200 beds]).

### Statistical analyses

First, patient and hospital characteristics were tabulated. Medians and interquartile ranges (IQR) were calculated for continuously coded variables, while frequencies and proportions were computed for categorical variables. Wilcoxon rank sum test, Pearson chi-square test and Fisher’s exact test were applied. Second, propensity score matching (PSM, ratio 1:2) was applied to age at admission, sex, race/ethnicity, CCI, hospital teaching status and hospital region and bedsize to maximally reduce the effect of bias and confounding. Third, multivariable logistic regression models (LRM) predicting adverse in-hospital outcomes and in-hospital mortality were fitted. All multivariable models relied on generalized estimating equations to further adjust for clustering [17–19]. 

All analyses and their reporting followed the NIS reporting guideline. Specifically, exact counts and associated proportions were not reported for samples with membership < 11 [20]. All tests were two sided, with a significance level set at *p* < 0.05. R software environment was used for statistical computing and graphics (R version 4.2.2; R Foundation for Statistical Computing, Vienna, Austria).

## Results

### Descriptive characteristics

Within the NIS (2008–2019), we identified 491 mRCC patients. Of those, 139 (28%) underwent RPCN vs. 352 (72%) OPCN (Table 1). No statistically significant differences were identified regarding patient characteristics (age, sex, race/ethnicity and CCI) as well as hospital characteristics (teaching hospital status, hospital bedsize and hospital region) between RPCN and OPCN patients. After 1:2 PSM, 139 of 139 (100%) RPCN and 278 of 352 (79%) OPCN patients were included in subsequent analyses. After PSM, differences between RPCN and OPCN were further reduced.

**Table 1:** Descriptive characteristics of 491 patients undergoing partial cytoreductive nephrectomy (PCN), stratified according to robotic or open surgical approach, before and after 1:2 PSM, within the nationwide inpatient sample (NIS) from 2008 to 2019

	Before 1:2 PSM			After 1:2 PSM		
Characteristic	Robotic PCN *n* = 139 (28%)^a^	Open PCN *n* = 352 (72%)^a^	*p*-value^2^	Robotic PCN *n* = 139 (33%)^a^	Open PCN *n* = 278 (67%)^a^	*p*-value^b^
Age	63 (55, 72)	63 (54, 71)	0.5	63 (55, 72)	64 (56, 71)	0.7
Male sex	95 (68%)	249 (71%)	0.6	95 (68%)	190 (68%)	1.0
Race/Ethnicity			0.4			0.8
Caucasians	98 (71%)	263 (75%)		98 (71%)	204 (73%)	
African Americans	14 (10%)	23 (7%)		14 (10%)	23 (8%)	
Others	27 (19%)	66 (19%)		27 (19%)	51 (18%)	
CCI			0.9			0.8
0	68 (49%)	176 (50%)		68 (49%)	144 (52%)	
1–2	52 (37%)	129 (37%)		52 (37%)	100 (36%)	
≥ 3	19 (14%)	47 (13%)		19 (14%)	34 (12%)	
Teaching hospital status	121 (87%)	312 (89%)	0.6	121 (87%)	249 (90%)	0.4
Bedsize			0.2			0.8
Large	95 (68%)	267 (76%)		95 (68%)	195 (70%)	
Medium	28 (20%)	50 (14%)		28 (20%)	49 (18%)	
Small	16 (12%)	35 (10%)		16 (12%)	34 (12%)	
Region			0.05			0.9
Midwest	35 (25%)	69 (20%)		95 (68%)	195 (70%)	
Northeast	34 (24%)	114 (32%)		28 (20%)	49 (18%)	
South	33 (24%)	104 (30%)		16 (12%)	34 (12%)	
West	37 (27%)	65 (18%)		95 (68%)	195 (70%)	

* CCI * harlson Comorbidity Index

^a^Median (Q1, Q3); n (%)

^b^Wilcoxon rank sum test; Pearson’s Chi-square test; Fisher’s exact test

### Rates of RPCN over the study span

**Fig. 1:**
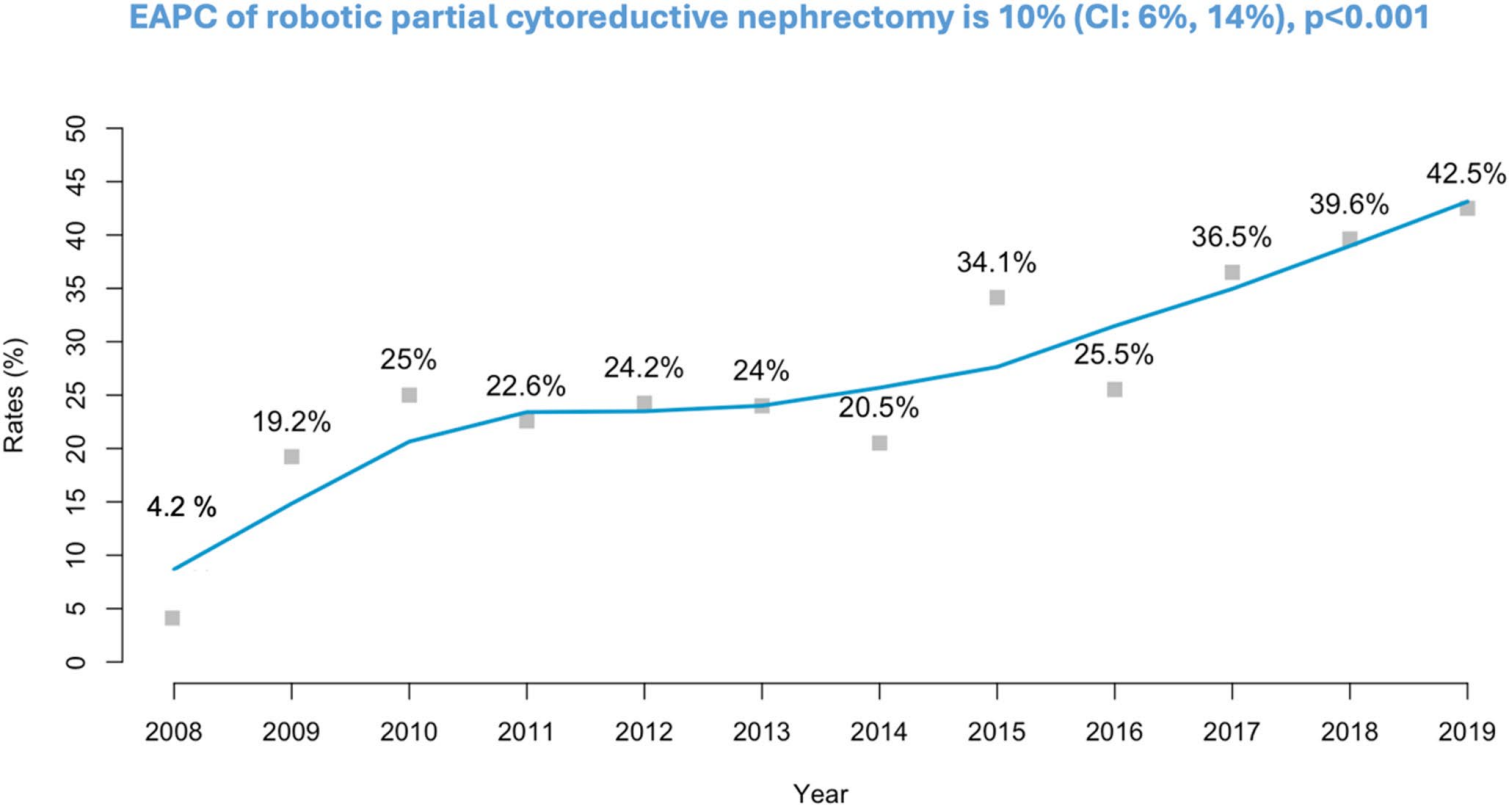
Annual rates of robotic partial cytoreductive nephrectomy within the National Inpatient Sample (2008–2019) with estimated annual percentage change

RPCN rates increased over the study span of 12 years (2008–2019) from 4.2% in the initial study year to 42.5% in the final study year (Fig. 1). This corresponded to an estimated annual percentage increase of 10% for RPCN (*p* < 0.001).

### Adverse in-hospital outcomes of RPCN vs. OPCN

After PSM, RPCN patients more frequently exhibited lower rates in four of 10 examined categories relative to their OPCN counterparts (Table 2). Specifically, RPCN patients exhibited lower rates of intraoperative complications (< 3 vs. 9%, *p* = 0.02), pulmonary complications (6 vs. 14%, *p* = 0.02), blood transfusions (< 5 vs. 14%, *p* = 0.004) and exhibited shorter median length of stay (2 vs. 4 days, *p* < 0.001). No statistically significant differences were observed in rates of overall, cardiac, vascular and gastrointestinal complications as well as median THC. Finally, no statistically significant differences in in-hospital mortality between RPCN and OPCN were recorded (all p-values ≥ 0.1).

**Table 2:** Adverse in-hospital outcomes (overall complications, intraoperative complications, postoperative complications and mortality) and length of stay in partial cytoreductive nephrectomy, stratified according to robotic vs. open approach, after 1:2 PSM

Characteristic	Robotic PCN *n* = 139 (33%)^a^	Open PCN *n* = 278 (67%)^1^	Δ%	*p*-value^b^	Multivariable OR* (95% CI)	*p*-value
Overall complications, n (%)	42 (30%)	104 (37%)	-7%	0.2	0.86 (0.54, 1.35)	0.5
Intraoperative complications, n (%)	< 11 (< 3%)	25 (9%)	**-6%**	**0.02**	**0.28 (0.08**,** 0.78)**	**0.02**
Pulmonary complications, n (%)	8 (6%)	38 (14%)	**-8%**	**0.02**	**0.34 (0.14**,** 0.74)**	**0.01**
Cardiac complications, n (%)	15 (11%)	18 (7%)	+ 4%	0.1	1.84 (0.81, 4.16)	0.14
Vascular complications, n (%)	< 11 (< 1%)	< 11 (< 3%)	-2%	0.3	0.20 (0.01, 1.27)	0.2
Gastrointestinal complications, n (%)	17 (12%)	30 (11%)	+ 1%	0.7	1.11 (0.56, 2.12)	0.8
Blood transfusions, n (%)	< 11 (< 5%)	37 (13%)	**-8%**	**0.004**	**0.25 (0.09**,** 0.59)**	**0.003**
Length of stay (median, IQR)	2 (2, 4)	4 (3, 6)	**-2 days**	**< 0.001**	**0.17 (0.09**,** 0.32)**	**< 0.001**
Tot. hospital charges $ (median, IQR)	68,977 (56,911, 95,541)	65,858 (43,836, 107,035)	+ 3,119 $	0.6	0.73 (0.54, 1.17)	0.2
In-hospital mortality	0 (0%)	< 11 (< 2%)	-<2%	0.3	/	/

* IQR * interquartile ranges

*Adjusted for surgical approach, age, sex, race/ethnicity, Charlson Comorbidity Index, hospital region, bed size, teaching hospital status

^a^Wilcoxon rank sum test; Pearson’s Chi-square test; Fisher’s exact test

### Multivariable logistic regression models

After PSM and additional multivariable adjustments for the aforementioned patient and hospital characteristics, RPCN independently predicted lower rates of adverse in-hospital outcomes in the same four of 10 examined categories (Table 2). Largest magnitude applied to median length of stay (OR 0.17, *p* < 0.001), followed by lower rates of intraoperative complications (OR 0.24, *p* = 0.02), lower rates of blood transfusions (OR 0.25, *p* = 0.003) and lower rates of pulmonary complications (OR 0.34, *p* = 0.01) in decreasing order.

## Discussion

Currently, it is unknown whether RPCN differs from OPCN when adverse in-hospital outcomes represent the endpoint of interest. To address this knowledge gap, we relied on the NIS database (2008–2019) and tested for the differences of adverse in-hospital outcomes between RPCN and OPCN. We made several noteworthy observations.

First, we identified 491 PCN patients. Of those, 139 (28%) underwent RPCN vs. 352 (72%) OPCN. No statistically significant differences were identified in patient characteristics such as age, sex, race/ethnicity and CCI. These observations indicate that robotic approach is offered to patients regardless of their age, sex, race/ethnicity and comorbidity status. Additionally, no statistically significant differences were recorded regarding hospital characteristics between RPCN vs. OPCN patients (teaching hospital status, hospital bedsize and hospital region). To the best of our knowledge, the current report validates absence of access barriers to RPCN based on patient and hospital characteristics.

Second, the current study cohort represents a first comparison between RPCN vs. OPCN. However, previous studies examined PCN in the context of mRCC without specific stratification according to robotic vs. open approach for the assessment of advisers in-hospital outcomes. These studies were based on PCN cohorts ranging from 46 to 237 patients from the Surveillance, Epidemiology and End Results (SEER) database [1, 2, 6, 21]. Additionally, two other studies by Hauser et al. (*n* = 654) and Lenis et al. (*n* = 381) reported on PCN patients from the National Cancer Database (NCDB) [5, 22]. All mentioned studies compared partial CN vs. radical CN regarding survival outcomes such as overall mortality (OM), cancer-specific mortality (CSM) and other-cause mortality (OCM). For example, Hauser et al. and Lenis et al. observed lower OM in PCN patients compared to their radical CN counterparts [5, 22]. Capitanio et al. and Tian et al. reported that, relative to radical CN, PCN is not associated with higher CSM [1, 6]. Finally, Mazzone et al. and Cano et al. observed lower OCM in patients undergoing PCN compared to their radical CN counterparts both in historical and contemporary cohorts [2, 21]. In consequence, relatively abundant literature exists regarding PCN. However, no previous study tested for differences between RPCN vs. OPCN and adverse in-hospital outcomes.

Absence of such study validates the need for the current study where such comparison is performed.

Third, regarding patient characteristics, the current study cohort is comparable to previously reported studies on PCN patients. Median age of the current study cohort is 63 years. This age distribution in in accordance with several previously studies addressing PCN patients, where patient ages ranged from 62 to 64 years [1, 2, 5, 6]. Additionally, male sex ranged from 68 to 76% in previous studies addressing PCN. This sex distribution is also comparable to the current study, where 70% were male [1, 2, 5, 6]. Finally, the distribution of race/ethnicity in the current study is also comparable to previously reported studies on PCN. Specifically, in previous reports, Caucasian patients accounted for 73 to 89% [1, 2, 5, 6] of study subjects vs. 68% of Caucasian patients in the current study cohort. Taken together, these observations validate the similarity of current study cohort patient characteristics relative to previous studies that addressed PCN patients. Of those, only one is larger than the current study cohort when absolute number of PCN patients is considered. Specifically, Hauser et al. who relied on the NCDB 2010–2017 identified 654 PCN patients.

Fourth, we examined rates of RPCN over the study years (2008–2019). Here, we observed a significant increase RPCN from 4.2% in the initial study year (2008) to 42.5% in the final study year (2019). This finding is in agreement with rates reported by Hauser et al. (NCDB 2010–2017) regarding the use of minimally-invasive PCN. Specifically, the authors observed an increase from 16 to 42% [5]. The upward trend of RPCN rates indicate that even in the setting of mRCC, an increasing proportion of patients receive PCN. Additionally, the use of robotic approach is also more frequently used. However, no other studies reported on these rates besides Hauser et al. and the current study. In consequence, these observations validate the pertinence of the current study that documented the important increase in RPCN.

Fifth, we tested for differences in adverse in-hospital outcomes in RPCN vs. OPCN patients within the PSM population. Specifically, RPCN patients exhibited lower rates in four of 10 adverse in-hospital categories, namely intraoperative complications (< 3 vs. 9%, *p* = 0.02), pulmonary complications (6 vs. 14%, *p* = 0.02), blood transfusions (< 5 vs. 14%, *p* = 0.004) and median length of stay (2 vs. 4 days, *p* < 0.001). In multivariable LRMs, RPCN independently predicted lower rates in the same four of 10 categories. Specifically, the largest protective effect was recorded regarding median length of stay (OR 0.17, *p* < 0.001), followed by lower intraoperative complications (OR 0.24, *p* = 0.02), lower rate of blood transfusions (OR 0.25, *p* = 0.003) and lower rate of pulmonary complications (OR 0.34, *p* = 0.01). Regarding in-hospital mortality, no mortality events were recorded in RPCN patients. Conversely, in OPCN less than 11 events (< 2%) of 278 patients were recorded. This difference between RPCN vs. OPCN in-hospital mortality rate was not statistically significant. Finally, multivariable analyses could not be completed due to insufficient numbers of observations. Taken together, these observations of in-hospital mortality indicate that the probability of in-hospital mortality is marginal at most, regardless of PCN approach. This finding is important to communicate at preoperative counseling when PCN is considered in mRCC patients. Unfortunately, the exact in-hospital mortality rate cannot be reported due to NIS reporting guideline. However, the provided estimates’ precision cannot be better than < 11 out of 278.

Sixth, the observations made regarding lower adverse in-hospital outcomes and shorter length of stay recorded at RPCN relative to OPCN are in agreement with previous reports where robotic vs. open approach were compared in the setting of other surgeries than PCN [12, 23–25]. Invariably, in all such previous analyses, robotic procedures were associated with fewer adverse in-hospital outcomes and shorter LOS compared to their open counterparts [12, 23–25]. Unfortunately, virtually all such previous analyses were based on the NIS that did not include stage and disease extent characteristics. In consequence, possibly several of such previous analyses, including the current one, could not account for potentially favorable tumor stage and grade selection biases within the robotic cohorts [12, 23–25]. Nonetheless, these findings validate the generalizability of the current study relative to previous reports [12, 23–25]. Unfortunately, no previous study tested for differences between RPCN vs. OPCN with respect to adverse in-hospital outcomes. In consequence, the current study results cannot be directly compared.

Taken together, the current observations indicate an increase in the use of RPCN in the setting of mRCC (4.2 to 42.5%). Additionally, no differences were identified in patient characteristics such as age, sex, race/ethnicity and comorbidity profile between RPCN and OPCN patients. This indicates that RPCN is offered equally frequently regardless of these patient characteristics. Second, the comparison between RPCN vs. OPCN regarding adverse in-hospital outcomes demonstrated a more favorable profile after RPCN than OPCN in four of 10 assessable categories. Moreover, no statistically significant differences were recorded in in-hospital mortality between RPCN and OPCN. It is of interest, that no mortality event was identified in 139 patients who underwent RPCN. Conversely, less than 11 of 276 (< 2%) mortality events in OPCN patients were recorded. This observation may be used to reassure PCN patients about marginal risk of in-hospital mortality regardless of robotic vs. open approach.

Despite its novelty, this study has limitations due to the retrospective nature of the NIS database. First, selection and reporting biases may be operational. Such biases applied to all previous studies that relied on the NIS [14, 15, 17, 19]. Second, despite the very large size of the NIS, the current cohort of RPCN vs. OPCN patients is very small and number of observations in some specific analyses could not be reported due to NIS reporting limitations. Specifically, real numbers could not be provided if < 11 observations occurred. Third, analyses on adverse in-hospital outcomes usually focus on 12–15 outcome categories [15, 17, 19, 23–30]. , However, in the context of PCN, too few observations were recorded in wound, infectious and genitourinary complications. In consequence, meaningful analyses could not be performed and these categories were excluded.

Fourth, the amount of detail included in the current analysis was also limited due to the nature of the NIS. Specifically, performance status, tumor characteristics such as location, size as well as tumor stage and tumor burden characteristics were not available. These variables represent determinants of surgical resection extent. In consequence, it is possible that the inclusion of these variables for purpose of adjustment would have reduced some of the apparent RPCN advantages. In that regard, it is plausible that RPCN patients harbored less extensive disease. The latter reduced the extent and duration of surgery required for most complete cytoreduction. Moreover, detailed information on variables such as timing of CN or type of systemic therapies were not provided within the NIS. However, these specific variables may play an important role in patients with mRCC regarding postoperative complications [30] as well as oncological outcomes [31, 32], as previous studies have shown. Specifically, the choice of certain systemic therapies may have led to a selection bias for robotic or open procedure that may influence the outcome of this study. Unfortunately, we could account for these variables, since they are not provided within the NIS. Finally, adverse outcomes that occurred after discharge could also not be included in the provided analyses and oncological outcomes such as progression free and overall survival could not be included due lack of these variables in the NIS. Despite those limitations, the current study provides a valuable insight into the potential benefits of RPCN relative to OPCN and quantifies the magnitude of RPCN advantages.

## Conclusion

RPCN outperformed OPCN in mRCC patients in four of 10 examined adverse in-hospital categories and reached independent predictor status for the same four of 10 examined categories. However, no differences regarding in-hospital mortality were recorded between RPCN and OPCN.

## Supplementary Information

Below is the link to the electronic supplementary material.

Supplementary Material 1

## Data Availability

No datasets were generated or analysed during the current study.
