# Supplementary material for: Robotic vs. open partial cytoreductive nephrectomy in metastatic renal cell carcinoma: adverse in-hospital outcomes
Source: World J Urol. 2025 Nov 11;43(1):687. doi: 10.1007/s00345-025-06080-8 (PMC12605386; doi:10.1007/s00345-025-06080-8)
Supplement: Supplementary file 1 — Supplementary Material 1 [file 345_2025_6080_MOESM1_ESM.docx]

**Supplementary Table 1:**

Code for metastatic sites

| ICD-9 | 197.0, 197.1, 197.2, 197.3, 197.4, 197.5, 197.6, 197.7, 197.8, 198.0, 198.1, 198.2, 198.3, 198.4, 198.5, 198.6, 198.7, 198.8, 198.81, 198.82, 198.89, 196.0, 196.1, 196.2, 196.3, 196.5,  196.6, 196.8, 196.9 |
| --- | --- |
| ICD-10 | C78, C780, C7800, C7801, C7802, C781, C782, C783, C7830, C7839, C784, C785, C786, C787, C788, C7880, C7889, C79, C790, C7900, C7901, C7902, C791, C7910, C7911, C7919, C792, C793, C7931, C7932, C794, C7940, C7949, C795, C7951, C7952, C796, C7960, C7961, C7962, C797, C7970, C7971, C7972, C798, C7981, C7982, C7989, C799, C77, C770, C771, C772, C773, C774, C775, C778, C779 |
